# A cascade model of mentorship for frontline health workers in rural health facilities in Eastern Uganda: processes, achievements and lessons

**DOI:** 10.1080/16549716.2017.1345497

**Published:** 2017-08-17

**Authors:** Judith Ajeani, Richard Mangwi Ayiasi, Moses Tetui, Elizabeth Ekirapa-Kiracho, Gertrude Namazzi, Rornald Muhumuza Kananura, Suzanne Namusoke Kiwanuka, Jolly Beyeza-Kashesya

**Affiliations:** ^a^ School of Medicine, Makerere University College of Health Sciences, Kampala, Uganda; ^b^ Department of Obstetrics and Gynaecology, Mulago National Referral Hospital, Kampala, Uganda; ^c^ Makerere University School of Public Health (MakSPH), Makerere University College of Health Sciences, Kampala, Uganda; ^d^ Epidemiology and Global Health Unit, Department of Public Health and Clinical Medicine, Umeå University, Umeå, Sweden

**Keywords:** MANIFEST — Maternal and Neonatal Implementation for Equitable Systems Study, Maternal health, mentorship, newborn, health workers, implementation science

## Abstract

**Background**: There is increasing demand for trainers to shift from traditional didactic training to innovative approaches that are more results-oriented. Mentorship is one such approach that could bridge the clinical knowledge gap among health workers.

**Objectives**: This paper describes the experiences of an attempt to improve health-worker performance in maternal and newborn health in three rural districts through a mentoring process using the cascade model. The paper further highlights achievements and lessons learnt during implementation of the cascade model.

**Methods**: The cascade model started with initial training of health workers from three districts of Pallisa, Kibuku and Kamuli from where potential local mentors were selected for further training and mentorship by central mentors. These local mentors then went on to conduct mentorship visits supported by the external mentors. The mentorship process concentrated on partograph use, newborn resuscitation, prevention and management of Post-Partum Haemorrhage (PPH), including active management of third stage of labour, preeclampsia management and management of the sick newborn. Data for this paper was obtained from key informant interviews with district-level managers and local mentors.

**Results**: Mentorship improved several aspects of health-care delivery, ranging from improved competencies and responsiveness to emergencies and health-worker professionalism. In addition, due to better district leadership for Maternal and Newborn Health (MNH), there were improved supplies/medicine availability, team work and innovative local problem-solving approaches. Health workers were ultimately empowered to perform better.

**Conclusions**: The study demonstrated that it is possible to improve the competencies of frontline health workers through performance enhancement for MNH services using locally built capacity in clinical mentorship for Emergency Obstetric and Newborn Care (EmONC). The cascade mentoring process needed strong external mentorship support at the start to ensure improved capacity among local mentors to provide mentorship among local district staff.

## Background

Despite concerted efforts over the last two decades to reduce maternal/newborn mortality and morbidity, Uganda failed to achieve the MDG targets [1]. Skilled birth attendance at birth has the potential to reduce maternal/newborn morbidity and mortality, but this has also fallen short of the country’s target. Poor quality of health services consistently feature as a reason for underutilisation of health facilities. Among the World Health Organization (WHO) six building blocks for health-systems strengthening (governance and financing, human resource for health, medical supplies and equipment, health-information systems, and service delivery), human resource for health (HRH) is a major contributor to strengthening health services [2]. Not only are adequate numbers of health workers needed, but those with the appropriate skills for the job demands.

A lot of attention has been paid to the inevitable increase of HRH numbers and health infrastructure expansion to cope with increasing patient loads, however the expansion of spaces within which health workers can be mentored to improve performance output has been neglected. Frontline health workers (health workers who offer maternal and newborn health services) have limited opportunities for continuous professional development beyond pre-service training. Access to experienced clinicians from whom junior staff can learn key skills and decision-making for improved maternal and newborn outcomes is limited [3]. In the absence of continuous medical education, skills acquired during pre-service training get eroded. Within the priorities of the Uganda National Minimum Health Care Package (UNMHCP), attention has been paid to the inadequate HRH numbers and expanding health infrastructure [4], but with little emphasis on how to develop the skills and competencies of HRH and better equip them to manage the rapidly changing work environment and population demands [5]. The WHO notes that simply increasing the numbers of workers is not enough; rather, scaling up educational programmes to produce multi-disciplinary service delivery teams is urgent and essential [1].

Furthermore, the shortage of health workers is compounded by the fact that their competencies, clinical experience and expectations are often poorly suited to the health needs of the populations they serve [6].

Although the value of refresher trainings for improving the quality of any health-care provision cannot be underscored, these trainings do not translate into improved service delivery if follow-up on-job enhancement is not done [4]. The effective application of the knowledge and skills learnt during trainings involves multidisciplinary teams working together to deliver health-care services [7]. Hence there is clearly a need to shift from traditional didactic training to results-oriented approaches [7]. Mentorship is one such approach that could bridge this gap. Uganda’s policy, as outlined in the Health Sector Strategic Plan III, advocates for a decentralised and cascading mentorship structure with higher-level facilities mentoring lower-level units [8].

Mentorship fosters ‘a supportive learning relationship between a caring individual who shares knowledge, experience and wisdom with another individual who is ready and willing to benefit from this exchange, to enrich their professional journey’ [9]. While the concept of clinical mentorship has worked very well in improving performance for antiretroviral therapy (ART), it is more difficult to apply when providing EmONC [10]. The fact that EmONC is very unpredictable and involves a complex set of skills and competencies to choose from, based on the presenting emergency, makes clinical mentorship for EmONC erratic and dynamic, instead of clear cut and precise as you would have with ART. This makes standardising the process quite challenging.

Different models have been used for clinical mentorship, ranging from field-based teams of mainly non-physician health workers, to highly skilled mentors (specialists in obstetrics/gynecology and pediatrics) and nurse mentors in India and Rwanda [4,11–13]. The basic principles are similar and include interaction between clinicians with knowledge and experience of working within the country’s health system and a lower-level cadre with limited supervision.

In Uganda, clinical mentorship for EmONC has been employed and resulted in improved maternal and newborn survival. However, the key gaps have been on sustainability issues over time. Work-based capacity-building approaches in the long term are expected to promote retention of health workers as well as improve service delivery [14]. Work-based training approaches have been implemented to strengthen professional capacity [15] and to increase health workers’ capacity to improve maternal, newborn and child health-service delivery.

A successful mentorship programme must address health-system issues in order to provide an enabling environment in which mentees can be observed at work and coached or mentored for better learning uptake to improve Maternal and Newborn Health (MNH) in the long term. The Maternal and Neonatal Implementation for Equitable Systems (MANIFEST) project [16] implemented in Uganda used an innovative cascading model approach to mentorship, using human resources for health by aligning the mentorship cascade to the existing health system, ensuring minimum disruption of staff roles and processes. The programme premised that the mentorship would cause a ripple effect through the existing health system with a higher likelihood of sustainability of the model beyond the project life.

Therefore, the aim of this study was to describe the mentorship process and highlight the achievements and lessons learnt from implementing a district-based mentorship programme with the aim of using these lessons for future scale-up of the mentorship process.

## Methods

### Study context and project area

The MANIFEST Project was implemented in three districts: Kamuli, Kibuku and Pallisa in Eastern Uganda from 2013 to 2015 [16]. The total population of the three districts was 1,106,100 [17]. The three districts had a total of 30 health facilities (27 Health Center IIs, two Health Center IVs and one general hospital). The project employed a comprehensive intervention that was comprised of two main components (community mobilisation and empowerment and health-systems strengthening) that have been described in detail elsewhere [16]. The health-systems component consisted of a three-pronged approach for improving the quality of maternal and newborn health services. The three main elements included strengthening leadership for maternal and newborn health (MNH) at district and facility level, motivation of health workers and mentorship. Mentorship was designed as a follow on from refresher trainings in Emergency Obstetrics and Newborn Care (EmONC), so as to maintain gains from the formal classroom teaching as well as strengthen competencies for EmONC for frontline health workers. This would be pivotal in delivering maternal and newborn health care (MNHC) equitably by enhancing access to high-quality care at the lowest referral health facilities, which was the overall goal of MANIFEST. Between 2013 and 2015, the MANIFEST project conducted a cascade mentorship model in 12 health facilities: four Comprehensive Emergency Obstetric and Newborn Care (CEmONC) and eight Basic Emergency Obstetric and Neonatal Care (BEmONC) health centres across three districts in rural Eastern Uganda. The cascade mentoring process was implemented in two phases: the initial mentoring of local mentors by central mentors and the actual mentoring process by local mentors. A total of 36 mentorship visits was carried out. This cascade mentoring process was driven by a theory of change that was modelled around the health-systems’ building blocks described by the World Health Organization [2].

### The cascade mentorship process

The main aspects of the mentorship process are summarised below.

#### Preparation for the first mentoring phase

A refresher training of health workers from August to October 2013 preceded the first mentoring phase. A total of 204 frontline health workers who offer maternal and newborn health services and members of the District Health Team members (69 in Kamuli, 52 in Kibuku and 83 in Pallisa) participated in the trainings. The training manual was adapted from the Advances in Labour and Risk Management tool (ALARM, 2008) and HBB + programmes recognised by Ministry Of Health for maternal and newborn care. Each training lasted five days and the content was tailored towards addressing the main causes of maternal and newborn death. Didactic lectures, group discussions and demonstrations were the main methods of training.

Pre-and post-written assessments were done as a means of assessing the knowledge gained from the training. Selection of local mentors was done by the district health teams based on criteria that included work experience, performance in the pre- and post-test, qualification and participation in maternal and newborn health activities within the district. Initially 12 local mentors were selected per district to make a total of 36 local mentors. However, only 16 mentors remained active throughout the project.

#### Training of district mentors

In October 2013, all the 36 selected potential mentors were invited for a further three-day orientation in mentorship. The training focused on the characteristics of a mentor, how to conduct a mentorship session, linkage of mentorship with supportive supervision, practical skills in emergency obstetric care, newborn resuscitation and management of intrapartum haemorrhage. There was a six-month lapse after the training and initiation of the first practical mentoring phase. Three to four mentoring teams were established per district. Each team was comprised of three to four members. Each team was assigned to a health centre and schedules and durations of mentoring sessions were drawn. A mentorship handbook was developed to guide the mentors through the mentorship process, in addition to a logbook that was to be used by the mentees to keep a record of activities done and any challenges encountered.

#### Mentorship of local mentors

Expert obstetricians and paediatricians (external mentors) conducted the mentoring of local mentors. The first mentoring phase lasted six months. The main purpose of initial mentoring sessions was to demonstrate how to conduct mentorship. The central mentors therefore led the first four sessions, while the local mentors led the last two. After each mentorship session, feedback was given, during which the mentorship process was assessed.

#### Evaluation of first mentorship phase

After the first mentorship phase, a qualitative evaluation was conducted to assess progress, identify problems and suggest solutions. The evaluation showed that health workers were more confident in the maternal-care component, but less confident in newborn care. Use of the mentee logbook was also noted to be particularly poor. These findings provided a basis for redesigning the second phase of mentorship. The mentee logbook was redesigned, with more focus on newborn care, by reconstituting the central team of mentors to include a pediatrician. Additionally, a decision was made to establish a resuscitation corner where there was none, in addition to treatment units for sick and pre-term babies in each of the referral facilities. The resuscitation corner consisted of a mattress laid on a hard surface, newborn resuscitation equipment, an oxygen source, source of warmth (blanket, overhead bulb), infection-control facilities and treatment algorithms. The spaces for pre-term babies and sick newborn care consisted of equipment, drugs and supplies for advanced care (intubation, incubator, continuous positive pressure ventilation, alternative feeding options and specialised drugs). It was also noted that some of the health workers who had been selected as mentors were not suitable to act as mentors. Therefore, a decision was made to re-evaluate them and to maintain only those considered capable for the second phase of mentorship.

#### Phase two mentorship

During the second phase of mentorship, the health centres designated for mentorship were expanded to include facilities that provided basic emergency obstetric and newborn care. Two additional health centres of level III were selected from each of the three mentorship districts. Selection of health centres was done in consultation with the district health office. Health centres which handled large volumes of maternal and newborn cases were also included.

The local mentors who had been involved in the mentorship activities were evaluated and categorised as ‘consistent and confident to mentor others’, ‘consistent but not confident to mentor others’ and ‘confident but constrained by hierarchical problems’. The mentors were evaluated in the broad areas of personal demeanour, attitude towards mentorship and confidence, suitability as a mentor in terms of knowledge/skills for EmONC, the actual conduct of mentorship, ability to teach and demonstrate skills for EmONC, ability to identify areas for mentorship at facility entry and during the visit, ability to give appropriate feedback, consistency and their own self-assessment as a mentor. The 16 mentors out of the 36 who were considered consistent and confident to mentor others continued with the mentorship process.

After six months, the final evaluation for the mentorship was conducted. This was conducted as part of the end-line evaluation for the programme. Table 1 provides a summary of the sessions that were covered during the mentorship visits.

**Table 1.** Key sessions covered during the mentorship visits.

Number	Topic/skills drill
1.	Partograph use ⱡ
2.	Newborn resuscitation ⱡ
3.	Care of the sick newborn
4.	Active management third stage of labour
5.	Postpartum haemorrhage (PPH) management
6.	Management of hypertensive disorders of pregnancy
7.	Intravenous fluid use in newborns ⱡ
8.	Use of misoprostol
9.	Manual vacuum aspiration use ⱡ
10.	Mixing jik/infection prevention ⱡ
11.	Postnatal care
12.	Pre-term baby care
13.	Management of convulsions in newborn
14.	Essential newborn care
15.	Safe, clean deliveries
16.	Obstructed labour management
17.	Post-abortion care
18.	Breastfeeding
19.	Record-keeping
20.	Supplies management
21.	Autoclave use ⱡ

**Key** ⱡ indicates that a knowledge and skills drill was done

Newborn resuscitation corners were established first in the higher-level centres such as hospital, HC IV and III. In Uganda, the district health system is organised in four tiers: health centres of level II (HCII); III (HCIII); IV (HCIV); and hospital. HC II provides ambulatory care including antenatal care and delivery; HC III provides, in addition, inpatient services and laboratory diagnostics. HC IV offers caesarean operations and blood transfusion in addition to level III care services, while the hospital is a district referral facility and provides oversight and leadership to the lower levels of care that are HC IV, III and II. The resuscitation corners were established in each of the referral centres in anticipation of referred pre-term and sick newborn babies.

### Data collection methods

Data for this paper were obtained through qualitative semi-structured interviews collected from July to October 2015. The interviews were designed to answer the specific research questions described in the introduction section. The data was collected until a point of saturation was reached at interview number 18; thereafter a decision was made to stop collecting any more data.

The key informants for this study were selected purposively based on their role within the district and the health facility as local mentors under the MANIFEST project. They included district health-team members, health facility in-charges and maternity in-charges of different facilities. Seven were from Kamuli, six from Pallisa and five from Kibuku districts, respectively. In terms of professional training, they included a mix of nurses (six), midwives (six), medical officers (two), clinical officers (two) and environmental officers (two). Their years of service ranged from three to 35, with an average of 13 years. Lastly, two-thirds [12] of these were female.

### Data management and analysis

The interviews were recorded using a digital recorder and transcribed verbatim to maintain their initial meaning. Data was analysed manually using the thematic analysis technique [18]. Four of the authors (JA, RMA, MT and RMK) independently read the interview transcripts and coded them in relation to the study objective. All the other authors then reviewed the codes through an iterative process, which yielded agreement on the codes to be further developed. From the codes, themes and subthemes were developed consistent with the objectives of the study through a similarly iterative process that involved all the authors. An example of how the themes were developed is summarised in Table 2. Lastly, through a process of reflection, the external mentors, who include some of the authors (JA and JBK), made self-reflections, which focused on lessons learnt for future implementation. The process of data analysis is summarised in the analysis train in Table 2.

**Table 2.** Example of the analysis process from text to themes.

Text	Codes	Sub-themes	Theme
We the staff have learnt many things, most didn’t know how to manage abortions and even just filling in partographs. With the mentorship, they are beginning to appreciate these skills more. Those days mothers were going away bleeding. But now everyone is able to do manual vacuum aspiration, they are able to quickly save life where there is an incomplete abortion. One thing I have seen is that the health workers are more motivated and now you find them at work. Even those who are on leave, sometimes they come to help. The health workers now cooperate with each other and they make sure that the patients are attended to. Like the other day, I could not make it but my colleague covered for me in maternity very well.	Learning new skills Managing abortions Filling in partographs Appreciating new skill sets Responding to patients’ needs Improved patient care Receiving life-saving skills Motivated Inspired Having team work Depending on others Being more available	Skills improvement Increased health-worker availability Enhanced teamwork	Increased health-worker productivity

Ethical approval to carry out the study was obtained from Makerere University School of Public Health Higher Degrees, Research and Ethics Committee. Before interviews, written informed consent was obtained from each key informant.

## Results

Here we take stock of the mentorship programme described above through four interrelated themes. These are: improved health-worker productivity; improved patient management; increased health-worker responsiveness; and, lastly, challenges encountered by the mentorship programme. The mentorship programme was viewed as having improved health workers’ productivity, which positively impacted on their patient management and responsiveness. Nonetheless this was not devoid of challenges from which we drew lessons as expounded in the discussion section.

### Increased health-worker productivity

Improved health-worker productivity stemmed from skills improvement in the area of maternal and newborn care and improved health-worker availability. Skills improvement was noted in the areas of partograph use and abortion management through manual vacuum extraction for the mothers. On the neonatal side, the main skill noted was resuscitation of newborns using a bag and mask to enhance their breathing. These skills were found to be essential for saving both the mothers and newborns. The quotes below illustrate the improvement in skills noted by the informants.

*‘We the staff, especially at the maternity, would not fill the partographs; we did not appreciate their importance and some of us did not know how to do it. With the mentorship, everything has changed. Nowadays we know that partographs are filled as a monitor for the mother and we take it seriously.’* (Health facility in-charge, Kamuli district).

*‘* *Those days mothers were going away bleeding. But now everyone is able to do manual vacuum aspiration; they are able to quickly save life where there is an incomplete abortion.’* (District health team member, Kibuku district).

Improved health-worker availability also contributed to increased health-worker productivity. According to the informants, the regular oversight from the mentorships created a sense of responsibility in them, which impacted on their availability. The health workers therefore reported to the health facilities earlier and reduced on their absenteeism. In addition, they were motivated by the anticipation of skills development in order to boost their competency and consequently work output. Similarly, the use of local mentors created a need to be exemplary, which boosted the availability of health facility in-charges and this consequently had a positive effect on the other health workers. The quote below illustrated how health-worker availability impacted on productivity.

*‘Before the in-charges used to be irregular at their health facilities. They used to spend, maybe only two days in a week at the facility. But now they schedule themselves to be at the facility every day, because they have a responsibility of mentoring others. Now you find more health workers at the health centers which is good for the patients.’* (District health-team member, Kamuli district).

Lastly, the availability was enhanced through teamwork and increased health-worker confidence. As noted earlier, the mentorship process involved working in teams to identify and resolve specific challenges. Teamwork and increased confidence in their ability to perform the required tasks created a positive effect on the health workers’ attitude towards work and enabled them to achieve more together. They were therefore more willing to provide support when it was required. For example, in one general hospital, some staff who were officially off-duty would come in for a few hours to help out in the maternity ward due to the increased facility deliveries during the project period. The quotes below demonstrated how teamwork and confidence-building enhanced health-worker productivity.

*‘Previously, a mother could complain about our availability. One said she came here four times and she could not find a midwife. But now after that MANIFEST mentorship program came in, I was like, “if the midwife is not there, I have to be there, because I can do most of the work that she does”. So I no longer allow mothers to go minus being attended to, even if it’s about delivery. We have been trained now; at least everyone can now conduct a safe normal delivery. Nurses, clinical officers, midwives – we all share the work now.’* (Health facility in-charge, Kibuku district).

*‘Mentoring has also given me skills and confidence; I remember one day I was sleeping – it was around 11 at night – then someone from Buseeta health facility called me that she had a complicated delivery. It was actually not complicated, I just told her what to do and the delivery was successful. I am now more confident about how to do my work because the mentorship really helped me to learn many things hands on, like I was not sure about how to handle retained placentas.’* (Maternity ward in-charge, Pallisa district).

### Improved patient management

With the improved skills, health workers became more competent and confident about managing patients. This was demonstrated through the reduced referrals and improvement in pre-referral treatment. According to the informants, some health workers were initially not confident about their skills levels and this often resulted in unnecessary referrals. This consequently led to overly burdened higher-level facilities, which resulted in a fall in the quality of care offered. The quote below illustrates the improved patient management at lower-level facilities.

*‘* *Actually, the number of babies we refer to hospital because of asphyxia is now limited, apart from those with completely poor APGAR score* [assessment of newborn wellbeing including heart rate, respiratory effort, muscle tone, response to stimulation, and skin coloration; a score of ten represents the best possible condition].*’* (Maternity ward in-charge, Pallisa district).

*‘Especially abortions which were referred to HC IV and it was increasing their workload, at least now at health center III, people are managing them, and the work load at HC IV has been reduced, which allows enough time to see the patients here.’* (Health facility in-charge, Kamuli district).

### Improved responsiveness among health workers

During the mentorship sessions, in-charges were encouraged to **identify local problems and to solve them and** this challenged them to be more responsive to their clients’ needs. In addition, it **fostered creativity**, which mainly entailed using existing resources to maximise output. Some of the major problems identified were inadequacy in supplies and equipment. Health workers learnt to use their own data for checking performance and estimating supplies needed for service delivery. They reduced the problem of stock-outs of supplies and drugs by improving their procurement procedures as illustrated in the quotes below.

*‘* *About the emergency kit, I have appreciated its importance more now. Before, we could just ignore and say that at our level (HC III), we do not need this. But when mentors came, we came to appreciate the importance of having magnesium sulphate, tetracycline and vitamin A for the babies, which has been so good for us.’* (Maternity ward in-charge, Kibuku district).

*‘* *We now analyze our data and performance, calculate our targets and we estimate the supplies that we may need. Previously we used to have stock outs of anti-malarias, but now because we know how many patients we see on a monthly basis, we are now able to stock enough anti-malarias. We still have challenges in other logistical supplies, but at least we use our data to quantify for medicines and supplies’* (Health facility in-charge, Kamuli district).

The exercise of challenging health workers to identify solutions to their challenges triggered them to think outside of the box. Facilities with extra equipment were encouraged to redistribute equipment such as bags and masks, equipment for helping newborns to breathe, among others, to other facilities. The facilities also designed a system of redistributing drugs that were in excess in some facilities or borrowing the drugs. The utilisation of equipment was also improved through efforts to identify unused equipment that had been locked up in the stores. For example, unused equipment such as manual vacuum aspirators and sterilisers were identified by some health facilities.

*‘* *Another thing we were able to identify was a sterilizer in our stores. It was just dormant in the store, and we were just (using) boiling equipment, which sometimes can be time consuming and unreliable. But right now when they [mentors] came in, at least we are using the sterilizer. They helped us identify it in our stores – can you imagine – and also taught us how to use it.’* (Maternity ward in-charge, Kamuli district).

‘*We didn’t know that we had manual vacuum aspirators in the store – can you imagine about 20 of them – and we were referring mothers; mothers were going away bleeding.’* (District health team member, Kibuku district).

*‘* *What helped us through the mentorships and supervision – we didn’t have this idea that you can borrow something from a facility. All we could do is to wait for National Medical Stores to bring and if it’s not there, you say it’s is not there. But the mentors came and challenged us. They said no, you can barrow from another facility if they have extra. We just need to pass through the district (to) find out which facility has what we are lacking.’* (Maternity in-charge, Pallisa district).

The health workers across the three districts were also challenged to set up special care service points for newborns. Consequently, newborn resuscitation corners were also set up in response to the high newborn deaths. Dedicated record books for managing newborn babies were also created in some facilities to improve record-keeping.

*‘* *Many children were dying because they were lacking oxygen, but as we talk now, through the MANIFEST project, we were able to set up a neonatal corner in every facility. Now our neonatal unit is functional in the hospital. Before then, children were dying; there is a time when 10 children died in the hospital. Now even if we have power load-shedding, we have created emergency lighting in the neonatal room through solar.’* (District health team member, Kamuli district).

### Challenges encountered by the mentorship programme

As noted in the introductory section of the results, despite the positive effects of the mentorship programme, some challenges were encountered. Here we describe three of the main challenges: [1] limited participation of medical doctors in the mentorship sessions; [2] heavy health workload; and [3] weak journal-keeping culture among health workers.

Most medical doctors in the three districts were often unable to join the mentorship sessions. This was related to three main reasons: one, they were generally less likely to be within the districts during the mentorship sessions. Compared to midwives, nurses or clinical officers, attracting and retaining medical officers in rural districts is more difficult. In most cases, those that are employed are also irregular at their work posts or work on a part-time basis. Secondly, the available ones expressed dissatisfaction at the areas of mentorship covered by the programme. They felt that the skill set being promoted was below their level of expertise and needed more challenging endeavours. Lastly, medical officers preferred that a mentorship that involved only medical doctors be organised for them, rather than being mentored with lower cadres. While the project team appreciated the need for separate mentorship sessions, it was not implemented mainly for two reasons: the mentorship programme was designed to promote collaboration and teamwork among different cadres, in order to resolve challenges. Secondly, the need for more medical doctors to play the role of local mentors was emphasised. The quote below demonstrated the limited participation of medical doctors.

*‘* *These doctors are difficult to get. First of all they are usually away; they just come like for one or two days in a week, as if they are constantly on call.’* (District health-team member, Pallisa district).

The second challenge was the heavy health-worker workload. Since the mentorship sessions were designed to happen at health facilities, the health workers were sometimes overwhelmed with balancing their work and the mentorship demands. This therefore implied that mentorship visits were often interrupted. The interruptions had a negative effect on their concentration span, which could have affected one’s level of competency, especially for skill sets that needed a heavy knowledge foundation. However, mentors endeavoured to provide support by working alongside the health workers during their work shift and by pre-scheduling visits on less busy days and limiting them to only two to three days per month in each district. Such a blend was found to be pragmatic, especially in the context of heavy workload, which was found to be common in these districts.

*‘Sometimes when the mentors come, we have a very long line of patients and balancing the two becomes difficult. But they (mentors) tried to work with us, so we have been learning while working, but sometimes it is hard.’* (Health facility in-charge, Kibuku district).

Lastly, a weak journal-keeping culture prevented consistent follow-up of health-worker learning. It was realised that health workers lacked a culture of writing. This made monitoring of learning needs difficult to follow up beyond the monthly two to three days of mentorship. According to the informants, while this could be a cultural issue, changing the culture could also be difficult, given the heavy workload of the health workers. To circumvent this challenge, assessments of hands-on skills during the mentorship sessions was undertaken, rather than basing on health-worker self-reports. The quote below illustrated the weak journal-keeping culture.

*‘Yeah, those journals, they are hard, you know for us we are not used to writing everything we do and also we can be very busy. And by the time the lines reduce, we are tired and we just keep forgetting.’* (Maternity ward in-charge, Kamuli district).

## Discussion

We demonstrated from this work that mentorship has the potential to improve MNH care provided by frontline staff if it is conducted consistently and with a clear focus [19]. Nonetheless, challenges of participation, workload and learning new forms of documentation exist. The areas that seemed to benefit most under MNH are partograph use, MVA use and newborn resuscitation/care of the sick newborn and pre-term babies. The referral process also improved by focusing on the principle and routine practice of pre-referral treatment. A similar study in Rwanda reported that mentorship yields very good results in sustaining high-quality service provision when used in ANC by nurse mentors [11]. In our discussion, we provide a deeper reflection on the cascade model of mentorship used and how this could have influenced the positive changes that we have observed.

In our study, we evaluated a cascade model for mentorship in EmONC using locally available human resources for health (HRH) supported by professional HRH in maternal and newborn care. We used a work-based approach, which allows discussion of problems within the local health system and identification of solutions that are contextually relevant [15].

The process began by selecting appropriate HRH that is able to carry out clinical mentorship and therefore demanded highly skilled and knowledgeable staff, preferably working in the areas of maternal and newborn care. We harnessed our mentors from in-service and through on-job training. The model we used is centered on existing health-system structures and policies, which make it easier to integrate into district health systems [20]. In contrast, the Rwandan model recruited and trained mentors earlier by decentralising pre-service training, which they were able to influence and tailor to meet the mentorship requirements [11]. Using locally available staff, however, yields restricted teams, since health expertise in MNH is limited in the rural areas and may compromise the quality of mentorship [21, 22]. However, it lowers the cost of travel and facilitation of an external team of mentors [23]. We experienced the problem of spending large resources in travel because most of our external mentors were based in the capital city. Use of external mentors from regional referral hospitals that are located closer to the districts may have been a better approach [24]. The Ethiopian study faced similar issues spending on transport for field-based teams for ART mentoring [12].

Although locally available HRH can be used for clinical mentorship in MNH, out of a wide base of health workers, only a few competent mentors will be found, hence building an adequate pool of district mentors may take time and cause burn-out on the part of the mentors [25]. One solution to this problem would be phased mentorships, taking on the facilities that need it the most and bringing these to an acceptable standard of care, and then recruiting new facilities as the old ones are weaned off. Such facilities would however still need frequent visits to maintain the quality of MNH care [26]. Key questions would then be: how long does it take to build acceptable standards of care and enforce this into routine behaviour for primary health workforce providing MNH?

We propose that skilled multidisciplinary teams must work together to address clinical (obstetric and neonatal issues) and management-related MNH issues. However, they must be involved right from the training stage and through the mentorship process. In this project, we had high-level skilled cadres (consultants with postgraduate training and several years of experience) involved. This led to skills impartation during the project life, hence the successes registered. The respect, authority and role modelling by the external mentors set the stage for the local mentors to take on the process.

Resource availability in terms of logistics, like fuel for transportation, staff allowances, and training equipment i.e. models for simulation, a continuum process for mentorship through phone or email, and time for quality mentorship is a key priority [27, 28]. Motivation of the local mentors through compensation of their time and ensuring that they were also mentored continuously by the external mentors and encouraged to refresh their own knowledge and skills in EmONC was also found to be critical [29].

Good leadership for MNH at district level improves the administration of health facilities and encourages innovation for MNH, leading to improvements in quality of care. Districts are therefore very instrumental in guiding the mentorship process and are a powerful force for advancing the programme. They must be engaged from the start and empowered to lead in order to sustain programme longevity [30, 31].

Most discussions on sustainability centred on the Ministry of Health advancing a policy that promotes mentorship, since this will attract budgetary allocations [32]. Districts were able in the short term to budget to modestly maintain mentorship, but expansion will clearly be limited due to lack of resources. The most sustainable path for mentorships seems to be through utilising health workers at higher-level facilities within each district [23]. There are, however, concerns about the limited numbers of suitable mentors that can be trained and who will consistently perform as such, given a voluntary dimension to the mentorship process [33]. Mid-level providers are more likely to become consistent mentors than more senior health cadres. This may be just as well, because the cadre of midwives gives the highest value for money to cause impact on the reduction of maternal and newborn mortality/morbidity [34].

However, having a sufficient number of health workers who possess the right skills for being a mentor is a prerequisite for a successful mentorship programme. The selection of district mentors was based on their performance at the refresher training, their qualification and their clinical experience. However, despite meeting the selection criteria, conducting mentorship depended on personal commitment. It was not surprising that building teams with highly skilled local mentors was very difficult. This was reflected in the high dropout rate (19/35) among those initially trained as mentors who either never mentored because they deemed themselves not capable, or dropped out along the way.

Another key pitfall for mentorship was the lack of structured tools to evaluate health worker practice and getting health workers to perform self-assessments so that mentorship can be tailored for their individual needs. Therefore, much of our evaluation was subjective, unlike the Indian model [9] that used case sheets and self-assessment tools, the latter being unpopular in our model.

### Strength and limitations

The cascade method of mentorship was a practical process that involved the districts’ leaderships right from the initial stage. The cascade method also capacitated the local mentors drawn from among the health workers. This early engagement with the stakeholders at the district level helps to create a sustainable system long after the project has wound up.

One of the limitations in this paper is that we could not strongly attribute the results only to our study, because there were other concurrent interventions in the district, such as supervision and community-level awareness [35, 36]. However, health workers who were part of the mentorship programme reported positive benefits with improved patient management and responsiveness. This favourable comment from health workers could be an indication that the mentorship had some positive effects. Nevertheless, more robust methodologies will be required to make attribution. During the final assessment of the mentorship programme, we did not interview women who utilised services in the intervention health centers, yet clients’ perspectives would have provided useful insights into benefits of the mentoring process among health workers.

Maintaining continuous interaction outside the scheduled visits was not possible. This was attributed to factors like limited logistical support, competing tasks/priorities among mentors and non-remuneration of the district mentors, leading to demotivation. Most of the mentorship evaluation was subjective. The mentees’ self-assessment tool, i.e. the logbook, was unpopular and never used by the mentees. One of the reasons that were given for not completing the logbook was that it was complicated. Deeper discussions revealed a fear of filling out the books as these would directly assess their work output. Monitoring and evaluating mentorship may be difficult and hard to standardise. In addition, a robust health-information system is necessary to clearly document the impact of mentorship.

## Conclusions

The cascade mentoring system was largely acceptable at the district level, but two critical bottlenecks should be addressed: the insufficient number of health workers in rural districts and compensation of mentors for the perceived additional workload. In addition to seniority, the criteria for selecting local mentors should include individual health-worker interest and passion for maternal and newborn care. For efficiency, polyvalent health workers who can address both clinical and management of MNH issues should be identified.

Internally placed staff can be used to mentor frontline health workers for improved MNH service delivery with good results. In order to attain sustainability of the mentorship process, districts need to be empowered to take charge of mentorship. There is no policy at the MOH regarding mentorship; therefore, these findings could guide further design and implementation of mentorship at the district local government level.

## Paper context

Mentorship among health workers is a motivating factor, which leads to higher quality of care and retention in service. This paper described experiences of mentorship for health workers in three resource-limited rural districts of Eastern Uganda. Results showed that mentorship is well accepted among health workers. However, mentorship was viewed as additional workload for the mentoring team. Future selection for mentors should consider the interests of the health worker in addition to seniority. Mentors should be polyvalent: capable of addressing clinical and managerial issues.
